# Effects of the Vertebral Artery Ostium/Subclavian Artery Angle on In-Stent Restenosis after Vertebral Artery Ostium Stenting

**DOI:** 10.1155/2021/5527988

**Published:** 2021-04-27

**Authors:** Hui Su, Shengyuan Yu, Chenglin Tian, Zhihua Du, Xinfeng Liu, Jun Wang, Xiangyu Cao

**Affiliations:** Department of Neurology, Chinese PLA General Hospital, First Medical Center, Beijing, China

## Abstract

**Methods:**

Between January 2016 and October 2018, sixty-four consecutive patients who underwent a total of 66 stenting procedures were screened for symptomatic and asymptomatic atherosclerotic VAOS. Of these patients, 57 had complete follow-up data. The baseline patient demographics and morphological features of the VAO were recorded. Potential factors influencing ISR, including conventional cerebrovascular disease risk factors, were assessed, together with outcome events including recurrent transient ischemic attack (TIA), stroke, and vascular-related mortality.

**Results:**

The average follow-up period was 13.2 ± 4.6 months. Technical success was achieved in all interventions. The degree of stenosis was reduced from 77.2 ± 6.1% to 13.7 ± 8.9% after the procedure. ISR was detected in eight treated vessels (14.0%) and occlusion in two (5.3%) arteries. Of the 57 patients, one had an ischemic stroke and 5 had TIAs. The angle of the VAO at the subclavian artery was associated with the risk of restenosis (preoperative, *P* = 0.04; postoperative, *P* = 0.02).

**Conclusions:**

Stenting is a feasible and effective treatment for VAOS. The angle of the VAO at the subclavian artery may contribute to the development of ISR.

## 1. Introduction

Atherosclerotic stenosis of the vertebral artery ostium (VAO), a recognized cause of ischemic stroke, accounts for 5.2–9% of posterior circulation ischemic strokes [1, 2]. Recent autopsy [3] and angiographic [4] studies have revealed VAO stenosis (VAOS) prevalence rates of 12.7% and 5.4%, respectively. Studies by The New England Medical Center Posterior Circulation Registry and Borgess Medical Center Vertebral Artery Ostium Stenting Registry found that atherosclerotic VAOS alone could explain posterior circulation ischemic stroke [5–7].

Currently, antiplatelet therapy is the first-line treatment for most posterior circulation strokes caused by VAOS or occlusion [8]. However, posterior circulation strokes are often refractory to medical treatment, which increases stroke recurrence [9]. Stent implantation for VAOS is a safe option for reducing the long-term risk of stroke [9, 10]; however, in-stent restenosis (ISR) is more common for stents placed at the origin of an artery than in those placed in the intracranial vertebral artery (VA) [11]. Moreover, the ISR rate of VAO stents is higher than that following carotid angioplasty and stenting [10, 12, 13]. Previous investigations have identified factors associated with ISR, including arterial anatomy, vessel diameter, coexisting carotid vascular occlusions, and medical management and stent type [14–17]. However, the effect of the VAO/subclavian artery angle in VAOS stent patients is seldom mentioned. This prospective, single-center study evaluated the incidence of and factors associated with ISR in 57 consecutive patients who underwent VAO stent placement.

## 2. Materials and Methods

### 2.1. Patient Characteristics

The study enrolled 64 consecutive patients treated with angioplasty and stenting for VAOS at our hospital between January 2016 and October 2018. Of these patients, 57 (53 males, 4 females; average age, 63.1 years; range: 44–81 years) had available follow-up data. All patients met the following inclusion criteria: symptomatic VAOS, defined as VAOS ≥ 50% with transient ischemic attack (TIA) or stroke in the region of the vertebral artery (VA); asymptomatic VAOS (≥70% with hypoplasia of the contralateral VA); satisfactory neurological function, defined by a modified Rankin Scale score ≤ 2 (if a minor stroke occurred, the procedure was performed at least 2 weeks after the onset of the most recent ischemic symptoms); and complete imaging and clinical data. Patients with nonatherosclerotic diseases, those with tandem or extensive lesions requiring the placement of more than one stent, and those who had undergone previous vertebral ostium interventions were excluded from the study. The degree of stenosis was measured on digital subtraction angiography using the North American Symptomatic Carotid Endarterectomy Trial method [18] and classified as <50%, 50–69%, or 70–99% stenosis, or occlusion.

The angiography and stent procedures were explained fully, and all patients provided informed consent.

### 2.2. Clinical and Angiographic Follow-Up

All patients were discharged the day after the procedure and prescribed aspirin (100 mg/day) and clopidogrel (75 mg/day) for at least 6 months. To minimize the risk of atherosclerosis, the patients were treated for atherosclerotic risk factors, including hypertension, hyperlipidemia, and diabetes. Follow-up involved telephone interviews and clinical visits 1, 3, 6, and 12 months after the procedure and yearly thereafter. Outcome events included TIA, stroke (affecting the anterior and posterior circulation), myocardial infarction, and death. There were no periprocedural complications. Cervical Doppler ultrasound was performed at 3, 6, and 12 months postprocedure. If ISR was suspected on Doppler examination according to the vertebral stenosis criteria (ostium site peak systolic velocity (PSV) ≥ 140 cm/s and PSV ratio (vertebral ostium stenosis PSV divided by intervertebral segment PSV) ≥ 2.1) [19], computed tomography angiography (CTA) or digital subtraction angiography was performed to detect ISR, which we defined as recurrent in − stent stenosis > 50%.

The mean follow-up duration was 13.2 ± 4.6 months. Of the eight patients with ISR, six (75.0%) had clinical events (cerebral infarction, *n* = 1; TIA, *n* = 5).

### 2.3. Stenting and Digital Subtraction Angiography Procedures

All procedures were performed by experienced interventional neurosurgeons. All patients received aspirin (100 mg) and clopidogrel (75 mg) daily for at least 5 days before the procedure. The procedures were performed under local anesthesia, and the patients were heparinized to achieve an activated clotting time of 150–200 s. A 6F guide catheter was placed into the subclavian artery at the VAO. Under roadmap guidance, a steerable 0.014-inch microwire was directed into the VAO through the stenotic lesion and placed in the distal cervical VA allowing adequate purchase for the stent to pass to the site of the stenosis. Care was taken to avoid plaque dislodgement. A balloon-expandable stent was selected according to the lesion length and diameter of the nondiseased distal segment of the VA. Then, the stent was carefully advanced into the stenosed segment under fluoroscopic guidance using an 11 cm image-intensifier diameter and X-ray pulse rate of 30 frames per second. The stent was deployed first by manual inflation of the balloon to achieve better controllability and then by controlled inflation to 13 atm using a manometer and deployed to protrude 1 to 2 mm into the subclavian artery to guarantee complete coverage of the stenotic lesion.

The stent type was selected according to the preference and experience of the neurosurgeon and the characteristics of the stenotic lesion. Three types of stents were used: an Express Vascular SD stent (Boston Scientific Co., Natick, MA, USA) was used in 54 procedures (94.7%), an Apollo stent (Microport, Shanghai, China) was used in two procedures (3.5%), and a Blue stent (Cordis, Miami, FL, USA) was used in one procedure (1.8%).

**Figure 1:**
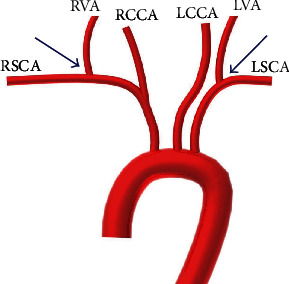
RVA: right vertebral artery; RCCA: right common carotid artery; LVA: left vertebral artery; LCCA: left common carotid artery; RSCA: right subclavian artery; LSCA: left subclavian artery. Vertebral artery ostium/subclavian artery angle (blue arrows).

The DSA images were imported into Digimizer Image Analysis Software V4.3.4 (MedCalc Software bvba, Ostend, Belgium) to obtain a 3D reconstruction of the prestent and poststent vertebral artery. The vertebral artery ostium/subclavian artery angle was defined the space between the vertibral artery and long axis of subclavian artery, and it was measured in degrees (Figure 1). The degrees were measured independently with the Digimizer Image Analysis Software V4.3.4 (MedCalc Software bvba, Ostend, Belgium) by two neuroradiologists.

### 2.4. Statistical Analyses

All statistical tests were performed using SPSS statistical software, version 25.0 (IBM Corp., Armonk, NY, USA). The one-sample Kolmogorov–Smirnov test was used to compare continuous variables. Normally distributed continuous variables are expressed as mean ± standard deviation. Differences between the ISR and non-ISR groups were assessed using Pearson's chi-squared test or Fisher's exact test for categorical data and Student's *t*-test for normally distributed continuous variables and the Mann–Whitney *U*-test for continuous skewed data. Kaplan–Meier curves and the log-rank test were used to show differences in ISR incidence between the groups. The hazard ratio (HR) and 95% CI of the different occlusion groups were assessed by Cox regression. All statistical tests were two-sided, with *P* values < 0.05 indicating statistical significance.

## 3. Results

Of the 64 patients screened, 7 did not receive follow-up. The clinical, demographic, and procedure characteristics of the remaining 57 patients are shown in Table 1 according to ISR status. Twenty-two patients also had a stenosis of the internal carotid artery. The study included 43 patients (75.4%) with symptoms, including dizziness in 32 cases (74.4%), focal weakness or focal sensory disturbance in 12 (27.9%), visual symptoms (diplopia or visual field defect) in 8 (18.6%), vertigo in 6 (14.0%), ataxia in 4 (9.3%), dysarthria in 3 (7.0%), and drop attacks in 1 (2.3%).

The procedure was technically successful in all cases, and no adverse events occurred before discharge. There was no association between ISR after VAO stent implantation and age, sex, risk factors, rate of stenosis, location of stenting, stent diameter, stent length, or VAO tortuosity (Table 1). Stents were implanted in the left (*n* = 35, 61.4%) and right (*n* = 22, 38.6%) VAO. The degree of VAOS was reduced from 77.2 ± 6.1% to 13.7 ± 8.9%. Angiographic follow-up was conducted 13.2 ± 4.6 months (range: 6–24 months) after the procedure. Among the 43 symptomatic patients, complete resolution of symptoms was observed in 35 (81.4%); the remaining 8 (18.6%) patients showed marked clinical improvement.

**Figure 2:**
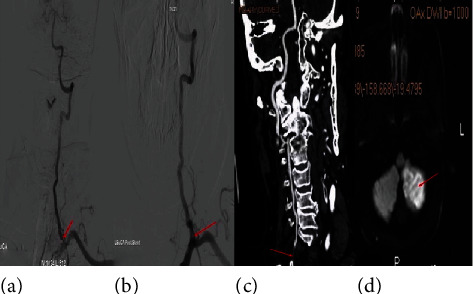
A 77-year-old male who presented with dizziness was admitted. His left subclavian arteriography shows severe stenosis of the vertebral artery ostium, and the vertebral artery ostium/subclavian artery angle was 93.9 degrees (a). After stenting of the left vertebral artery, ostium stenosis showed excellent dilatation of the lesion; then, the vertebral artery ostium/subclavian artery angle was 72.0 degrees (b). About ten months later, he was readmitted to the hospital because of dizziness reattack. His neck CTA showed left vertebral artery ostium occlusion (c). Brain MRI in DWI sequence showing left cerebellum restricted diffusion (d). Arrow in (a), stenosis site; arrow in (b), stent site; arrow in (c), occlusion site; arrow in (d), left cerebellum ischemic lesion.

**Figure 3:**
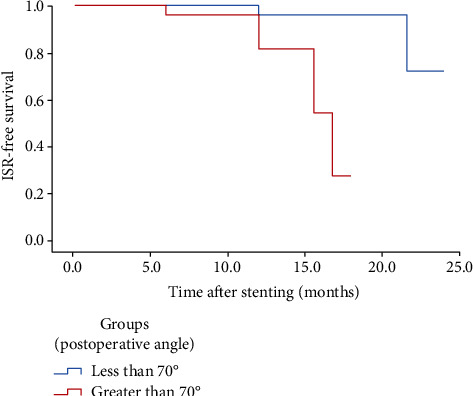
Kaplan-Meier estimates of cumulative ISR rate among patients with stenting in VAO.

The severity of stenosis on follow-up angiography exceeded 50% in eight cases (14%), two (3.5%) of which were shown by CTA to be occluded at 6 and 10 months (Figure 2). Six of the restenosis events were symptomatic: five patients had recurrent TIAs after a mean of 9.9 months (range: 6–12 months), and one patient had an acute cerebellar infarction at 12 months (Figure 2); he died of a heart attack 21 months after stent placement. The angle of the VAO at the subclavian artery exceeded 70° in six patients. Comparison of the ISR and non-ISR groups during follow-up revealed that the angle of the VAO at the subclavian artery (preprocedure, 63.8 ± 18.5°; postprocedure, 67.3 ± 15.7°) was associated with ISR (preoperative *P* = 0.04; postoperative *P* = 0.02) (Figure 3). There were no associations among mean age, sex, and risk factors (Table 1).

## 4. Discussion

We found that VAOS was more common in males than females. More than 50% ISR was found in 8 (14%) patients, most of whom were symptomatic. In contrast to other studies [20], no TIA, stroke, or death occurred within 30 days of the intervention in our series. To our knowledge, this is the first report that the angle of the VAO at the subclavian artery, both before and after stenting, can affect ISR.

Current treatments for VAOS include medical intervention, open surgery, percutaneous transluminal angioplasty, and stenting. All of the treatment options have advantages and drawbacks, which differ depending on the patient's race, the medications and stent types used, and the follow-up period. Motarjeme et al. performed the first VAO angioplasty in 1981 [21], and Storey et al. was the first to report VAO stent placement in 1996 [22]. The risk of extracranial VA stenting is lower than that of intracranial VA stenting [11, 15]. Moreover, despite a strict drug regimen after VAO stenting, ISR remains a frequent complication [23]. Although stenosis may be asymptomatic in patients with good collaterals, ISR is not always benign, and some patients who present with ischemic symptoms may ultimately require secondary stent placement.

In our study, ISR was observed in 14% of the patients after a mean follow-up of 13.2 ± 4.6 months. The reported rate of restenosis after endovascular treatment for VAOS can reach 67% [13, 24], which is higher than in our series. The risk factors for ISR remain unclear. Factors previously shown to contribute to ISR include age, diabetes mellitus, smoking, hyperlipidemia, small diameter stent (≤4 mm), longer stent length, tortuous V1 segment, long stenosis (>10 mm), contralateral VA hypoplasia, and contralateral VA occlusion at the time of stenting [17, 25–34]. The VAO has a high elastin concentration, which may increase the risk of stent recoil, kinking, and deformation, and contribute to ISR [35, 36].

Our findings confirm that the VAO/subclavian artery angle is associated with ISR after VAO stent placement. The angle of the VAO at the subclavian artery was 70–90° in 62.5% of the patients with ISR, which was significantly different from that of the non-ISR group. Local hemodynamic flow patterns change with vessel angle; the greater the angle, the higher the wall shear stress (WSS), which ultimately leads to ISR. A previous study found that high WSS at the junction of the VAO and subclavian artery markedly affected local blood flow [37]. A WSS of 2.0 Pa is sufficient to maintain the structure of arterial vessels. Low (<0.4 Pa) and high (>40 Pa) WSS contribute to ISR via different mechanisms [38, 39]. Moreover, flow velocity and pressure may influence the development of ISR. Several recent studies have investigated the effect of VAO stent location in an attempt to prevent ISR. One study found that implanting the stent 1 mm into the subclavian artery produced the smallest decrease in blood flow and WSS [34]. Although we successfully placed the proximal VA stent into the subclavian artery to a depth of 1–2 mm in all of our patients, ISR developed in several cases. Therefore, further investigation of stent type and placement in relation to the VAO/subclavian artery angle is warranted.

## 5. Study Limitations

The retrospective design, small sample size, and relatively high rate of loss to follow-up (10.9%) were the major study limitations of this study. Furthermore, the majority of the clinical follow-up data were obtained using noninvasive imaging techniques, which may have affected the assessment of ISR.

## 6. Conclusion

Our findings suggest that the VAO/subclavian artery angle significantly affects the incidence of ISR. Future investigations of ISR prevention through intensive medical treatment should focus on fluid–structure interactions and appropriate stent selection. Furthermore, studies with larger sample sizes and longer follow-ups are needed.

## Figures and Tables

**Table 1:** Demographic and clinical summary of vertebral artery ostium in patients with and without in-stent restenosis at follow-up.

Variable	ISR ≤ 50% (*n* = 49)	ISR > 50% (*n* = 8)	*P*	HR (95% CI)
Mean age (y)	63.2 ± 9.7	62.3 ± 9.8	0.54	0.64 (0.15-2.71)
Male (*n* (%))	46 (93.9)	7 (87.5)	0.64	0.58 (0.06-5.89)
Hypertension (*n* (%))	32 (65.3)	4 (50.0)	0.55	0.64 (0.14-2.84)
Diabetes (*n* (%))	15 (30.6)	2 (25.0)	0.78	1.30 (0.20-8.51)
Hyperlipidemia (*n* (%))	6 (12.2)	6 (75.0)	0.15	3.89 (0.60-25.13)
Coronary artery disease (*n* (%))	16 (32.7)	1 (12.5)	0.62	0.58 (0.07-5.10)
Smokers (*n* (%))	33 (67.3)	4 (50.0)	0.52	0.60 (0.12-2.92)
History of alcohol use (*n* (%))	26 (53.1)	3 (37.5)	0.38	0.51 (0.12-2.28)
Symptoms (*n* (%))				
TIA	34 (69.4)	5 (62.5)	0.45	0.37 (0.03-5.11)
Stroke	3 (6.1)	1 (12.5)	0.30	0.27 (0.02-3.20)
No symptoms	12 (24.5)	2 (25.0)	0.58	0.65 (0.16-3.10)
Contralateral vertebral				
Stenosis or occlusion (*n* (%))	13 (26.5)	2 (25.0)	0.50	0.50 (0.08-3.44)
Preoperative stenosis rate (%)	77.5 ± 6.0	75.0 ± 6.2	0.06	0.13 (0.01-1.08)
Residual stenosis (%)	13.7 ± 9.2	13.2 ± 7.1	0.34	0.15 (0.00-7.92)
Location of stenting (*n* (%))			0.84	1.22 (0.18-8.40)
LVAO	30 (61.2)	5 (62.5)		
RVAO	19 (38.8)	3 (37.5)		
Stent diameter (mm)	4.7 ± 0.8	4.8 ± 0.8	0.17	0.28 (0.05-1.72)
Stent length (mm)	15.7 ± 2.6	15.1 ± 1.4	0.57	3.11 (0.06-167.04)
Vessel tortuosity (*n* (%))	6 (12.2)	2 (25.0)	0.09	0.04 (0.00-1.80)
Preoperative angle (°)			0.04	0.19 (0.04-0.96)
≥70°	16 (72.7)	6 (27.3)		
<70°	33 (94.3)	2 (5.7)		
Postoperative angle (°)			0.02	12.11 (1.40-105.04)
≥70°	19 (76.0)	6 (24.0)		
<70°	30 (93.8)	2 (6.2)		

ISR: in-stent restenosis; TIA: transient ischaemic attack; LVAO: left vertebral artery origin; RVAO: right vertebral artery origin.

## Data Availability

If necessary, we can provide the crude data via the E-mail of corresponding author and data available in supplementary files.

## Supplementary Materials

Supplementary 1 57 patients' baseline characteristics at the time of the procedure.

Supplementary 2 Clinical characteristics of 57 patients (1).

Supplementary 3 Clinical characteristics of 57 patients (2).

Supplementary 4 Clinical characteristics of 57 patients (3).
