# Supplementary material for: Effects of the Vertebral Artery Ostium/Subclavian Artery Angle on In-Stent Restenosis after Vertebral Artery Ostium Stenting
Source: Biomed Res Int. 2021 Apr 27;2021:5527988. doi: 10.1155/2021/5527988 (PMC8101481; doi:10.1155/2021/5527988)
Supplement: Supplementary 1 — 57 patients' baseline characteristics at the time of the procedure. [file 5527988.f1.docx]

57 patients baseline characteristics at the time of the procedure

| No | sex | age | DM | HP | HL | CI | HD | Smoke | Drinking |
| --- | --- | --- | --- | --- | --- | --- | --- | --- | --- |
|  |  |  |  |  |  |  |  |  |  |
| 1 | 1 | 47 | 0 | 0 | 0 | 0 | 0 | 1 | 0 |
| 2 | 1 | 65 | 0 | 1 | 0 | 1 | 0 | 1 | 1 |
| 3 | 1 | 55 | 0 | 1 | 0 | 0 | 0 | 1 | 1 |
| 4 | 1 | 79 | 0 | 0 | 0 | 1 | 0 | 1 | 1 |
| 5 | 1 | 81 | 1 | 0 | 0 | 1 | 0 | 0 | 0 |
| 6 | 1 | 64 | 0 | 1 | 0 | 0 | 0 | 1 | 1 |
| 7 | 1 | 58 | 0 | 0 | 0 | 0 | 0 | 1 | 0 |
| 8 | 1 | 61 | 0 | 0 | 0 | 0 | 1 | 1 | 0 |
| 9 | 1 | 54 | 1 | 0 | 0 | 0 | 0 | 1 | 0 |
| 10 | 1 | 65 | 1 | 1 | 0 | 1 | 0 | 0 | 1 |
| 11 | 1 | 46 | 0 | 1 | 0 | 0 | 1 | 0 | 0 |
| 12 | 1 | 64 | 1 | 1 | 1 | 0 | 1 | 0 | 0 |
| 13 | 1 | 71 | 0 | 0 | 0 | 1 | 0 | 1 | 0 |
| 14 | 1 | 59 | 1 | 1 | 0 | 0 | 0 | 1 | 1 |
| 15 | 1 | 69 | 1 | 1 | 0 | 0 | 1 | 1 | 0 |
| 16 | 1 | 63 | 0 | 1 | 1 | 0 | 0 | 0 | 0 |
| 17 | 1 | 60 | 1 | 1 | 0 | 0 | 1 | 1 | 1 |
| 18 | 1 | 58 | 1 | 1 | 0 | 0 | 1 | 1 | 0 |
| 19 | 1 | 48 | 1 | 1 | 0 | 1 | 0 | 1 | 1 |
| 20 | 1 | 67 | 0 | 1 | 0 | 0 | 0 | 0 | 1 |
| 21 | 1 | 60 | 0 | 0 | 0 | 0 | 0 | 0 | 1 |
| 22 | 1 | 70 | 0 | 1 | 0 | 0 | 0 | 1 | 1 |
| 23 | 1 | 70 | 0 | 0 | 0 | 0 | 0 | 1 | 1 |
| 24 | 1 | 76 | 0 | 1 | 0 | 0 | 0 | 1 | 1 |
| 25 | 1 | 70 | 0 | 1 | 0 | 0 | 1 | 1 | 1 |
| 26 | 1 | 65 | 1 | 1 | 0 | 0 | 0 | 1 | 1 |
| 27 | 2 | 60 | 1 | 0 | 0 | 1 | 1 | 0 | 0 |
| 28 | 1 | 67 | 0 | 1 | 0 | 0 | 0 | 1 | 1 |
| 29 | 1 | 57 | 0 | 1 | 0 | 0 | 0 | 1 | 1 |
| 30 | 1 | 63 | 0 | 0 | 1 | 1 | 0 | 0 | 0 |
| 31 | 1 | 73 | 0 | 0 | 0 | 0 | 1 | 1 | 0 |
| 32 | 1 | 79 | 0 | 1 | 0 | 0 | 0 | 1 | 1 |
| 33 | 1 | 66 | 0 | 1 | 0 | 0 | 0 | 0 | 0 |
| 34 | 1 | 57 | 0 | 0 | 1 | 0 | 0 | 0 | 0 |
| 35 | 1 | 47 | 1 | 1 | 0 | 0 | 0 | 0 | 0 |
| 36 | 1 | 36 | 1 | 1 | 0 | 0 | 0 | 1 | 0 |
| 37 | 2 | 58 | 0 | 0 | 0 | 0 | 1 | 0 | 0 |
| 38 | 1 | 63 | 0 | 0 | 0 | 0 | 0 | 0 | 0 |
| 39 | 1 | 44 | 0 | 0 | 0 | 0 | 0 | 1 | 1 |
| 40 | 1 | 58 | 0 | 1 | 1 | 1 | 0 | 1 | 0 |
| 41 | 1 | 70 | 0 | 1 | 1 | 0 | 1 | 1 | 1 |
| 42 | 1 | 66 | 0 | 0 | 0 | 0 | 1 | 0 | 0 |
| 43 | 1 | 62 | 0 | 1 | 0 | 0 | 0 | 1 | 1 |
| 44 | 1 | 55 | 0 | 1 | 0 | 0 | 1 | 1 | 0 |
| 45 | 1 | 61 | 1 | 1 | 0 | 0 | 1 | 1 | 1 |
| 46 | 1 | 52 | 0 | 1 | 0 | 1 | 0 | 1 | 1 |
| 47 | 1 | 73 | 0 | 1 | 0 | 0 | 0 | 0 | 0 |
| 48 | 1 | 68 | 1 | 1 | 0 | 0 | 0 | 0 | 1 |
| 49 | 1 | 73 | 0 | 1 | 1 | 0 | 1 | 1 | 1 |
| 50 | 2 | 56 | 0 | 0 | 0 | 0 | 0 | 0 | 0 |
| 51 | 1 | 57 | 1 | 1 | 0 | 0 | 0 | 1 | 1 |
| 52 | 1 | 79 | 0 | 0 | 0 | 0 | 0 | 1 | 1 |
| 53 | 1 | 71 | 0 | 0 | 0 | 0 | 1 | 1 | 0 |
| 54 | 1 | 75 | 0 | 1 | 0 | 1 | 0 | 1 | 1 |
| 55 | 1 | 59 | 0 | 1 | 1 | 0 | 0 | 1 | 1 |
| 56 | 2 | 77 | 1 | 1 | 0 | 1 | 0 | 0 | 0 |
| 57 | 1 | 69 | 0 | 0 | 0 | 0 | 1 | 0 | 0 |
