# Supplementary material for: Effects of the Vertebral Artery Ostium/Subclavian Artery Angle on In-Stent Restenosis after Vertebral Artery Ostium Stenting
Source: Biomed Res Int. 2021 Apr 27;2021:5527988. doi: 10.1155/2021/5527988 (PMC8101481; doi:10.1155/2021/5527988)
Supplement: Supplementary 2 — Clinical characteristics of 57 patients (1). [file 5527988.f2.docx]

Clinical Characteristics of 57 Patients (1)

| No | Stenosis  (Contr-VA) | Symptoms  (Pre-stent) | AA inhibition | ADP inhibition | Symptoms  (Post-stent) |
| --- | --- | --- | --- | --- | --- |
|  |  |  |  |  |  |
| 1 | 0 | 1(Yes) | 90 | 76 | 1(Yes) |
| 2 | 0 | 1 | 87 | 56 | 1 |
| 3 | 0 | 1 | 45 | 41.1 | 0(No) |
| 4 | 0 | 1 | 0 | 83.2 | 0 |
| 5 | 1 | 1 | 96.3 | 42 | 0 |
| 6 | 0 | 1 | 92.2 | 48.6 | 1 |
| 7 | 0 | 1 | 99.1 | 74.4 | 0 |
| 8 | 0 | 1 | 95.6 | 75.5 | 0 |
| 9 | 0 | 1 | 96.5 | 88.7 | 0 |
| 10 | 0 | 1 | 95 | 56.6 | 1 |
| 11 | 0 | 1 | 80.2 | 55.5 | 1 |
| 12 | 0 | 1 | 88.1 | 28.7 | 1 |
| 13 | 0 | 1 | 96.6 | 68.1 | 0 |
| 14 | 0 | 1 | 94.3 | 52.2 | 2 |
| 15 | 0 | 1 | 100 | 100 | 0 |
| 16 | 0 | 1 | 68.8 | 57.1 | 0 |
| 17 | 0 | 1 | 100 | 71.3 | 1 |
| 18 | 0 | 1 | 91 | 22.6 | 0 |
| 19 | 0 | 1 | 76.2 | 58 | 1 |
| 20 | 0 | 0(No) | 96.4 | 48.1 | 0 |
| 21 | 0 | 1 | 0.6 | 70 | 0 |
| 22 | 0 | 0 | 97.2 | 84.2 | 0 |
| 23 | 0 | 0 | 100 | 48 | 0 |
| 24 | 0 | 0 | 90.4 | 96.9 | 0 |
| 25 | 0 | 1 | 90.4 | 26.3 | 0 |
| 26 | 0 | 0 | 89.1 | 15.9 | 0 |
| 27 | 0 | 1 | 86.5 | 45.3 | 0 |
| 28 | 0 | 1 | 100 | 67.8 | 0 |
| 29 | 0 | 1 | 95.6 | 45.5 | 0 |
| 30 | 0 | 1 | 99.4 | 60.7 | 1 |
| 31 | 0 | 1 | 95.2 | 34.3 | 0 |
| 32 | 0 | 1 | 57.8 | 44 | 0 |
| 33 | 0 | 1 | 98.3 | 40.4 | 0 |
| 34 | 0 | 0 | 98.3 | 45.7 | 0 |
| 35 | 1 | 0 | 100 | 38.6 | 0 |
| 36 | 1 | 1 | 95.5 | 68 | 0 |
| 37 | 0 | 1 | 48.8 | 1.9 | 0 |
| 38 | 1 | 1 | 53.7 | 63.1 | 1 |
| 39 | 0 | 0 | 99.8 | 49.5 | 0 |
| 40 | 0 | 0 | 68.2 | 30.5 | 0 |
| 41 | 0 | 1 | 60.7 | 48 | 0 |
| 42 | 1 | 1 | 94.9 | 70.4 | 1 |
| 43 | 0 | 0 | 98.5 | 26.6 | 0 |
| 44 | 1 | 0 | 92.8 | 40.7 | 0 |
| 45 | 0 | 1 | 90 | 59.1 | 0 |
| 46 | 1 | 1 | 93.8 | 97 | 0 |
| 47 | 0 | 1 | 100 | 52.9 | 0 |
| 48 | 0 | 1 | 91.6 | 52.8 | 1 |
| 49 | 0 | 1 | 85.7 | 42.7 | 0 |
| 50 | 0 | 1 | 85.4 | 33.5 | 0 |
| 51 | 0 | 0 | 95.9 | 52.4 | 0 |
| 52 | 2 | 1 | 78 | 45 | 0 |
| 53 | 0 | 0 | 95.7 | 46.3 | 0 |
| 54 | 0 | 1 | 85.6 | 28.5 | 0 |
| 55 | 2 | 1 | 97.9 | 26.4 | 0 |
| 56 | 0 | 0 | 51.7 | 48.4 | 0 |
| 57 | 0 | 1 | 96.6 | 97.6 | 0 |
