# Supplementary material for: Effects of the Vertebral Artery Ostium/Subclavian Artery Angle on In-Stent Restenosis after Vertebral Artery Ostium Stenting
Source: Biomed Res Int. 2021 Apr 27;2021:5527988. doi: 10.1155/2021/5527988 (PMC8101481; doi:10.1155/2021/5527988)
Supplement: Supplementary 3 — Clinical characteristics of 57 patients (2). [file 5527988.f3.docx]

Clinical Characteristics of 57 Patients (2)

| No | stent diamete  （mm） | Stent length（mm） | angle  (Pre-stent) | angle  (Post-stent) |
| --- | --- | --- | --- | --- |
|  |  |  |  |  |
| 1 | 5 | 15 | 56.99 | 52.3 |
| 2 | 4 | 15 | 33.69 | 59.83 |
| 3 | 4 | 15 | 46.48 | 74.61 |
| 4 | 4 | 15 | 41.69 | 37.45 |
| 5 | 4 | 15 | 50.16 | 65.94 |
| 6 | 5 | 15 | 49.24 | 48 |
| 7 | 5 | 15 | 48.85 | 48.62 |
| 8 | 4 | 19 | 34 | 43.71 |
| 9 | 4 | 15 | 57.47 | 58.19 |
| 10 | 6 | 14 | 53.97 | 63.68 |
| 11 | 5 | 15 | 46.2 | 41.82 |
| 12 | 4 | 15 | 39.35 | 45 |
| 13 | 6 | 14 | 40.77 | 45.39 |
| 14 | 6 | 14 | 46.85 | 43.9 |
| 15 | 6 | 14 | 36.97 | 46.16 |
| 16 | 4 | 19 | 29.73 | 44.84 |
| 17 | 4 | 15 | 50.47 | 66.59 |
| 18 | 5 | 15 | 38.69 | 60.63 |
| 19 | 4 | 15 | 57.32 | 74.19 |
| 20 | 4 | 15 | 57.07 | 92.18 |
| 21 | 4 | 15 | 56.77 | 56 |
| 22 | 4 | 15 | 40.57 | 90 |
| 23 | 7 | 30 | 48.45 | 68.42 |
| 24 | 5 | 15 | 40.09 | 70.39 |
| 25 | 5 | 15 | 60.58 | 72.15 |
| 26 | 5 | 15 | 62.97 | 66.68 |
| 27 | 4 | 15 | 67.83 | 48.28 |
| 28 | 6 | 19 | 63.48 | 78.17 |
| 29 | 4 | 15 | 60.77 | 70.23 |
| 30 | 3.5 | 13 | 67.59 | 78.67 |
| 31 | 5 | 15 | 68.9 | 78.2 |
| 32 | 5 | 15 | 65.84 | 63.13 |
| 33 | 4 | 15 | 65.25 | 46.67 |
| 34 | 5 | 15 | 65.57 | 82.26 |
| 35 | 5 | 15 | 64.32 | 66.76 |
| 36 | 6 | 14 | 85.69 | 90.83 |
| 37 | 4 | 15 | 74.48 | 62.15 |
| 38 | 6 | 14 | 72.35 | 63.47 |
| 39 | 5 | 19 | 106.93 | 94.52 |
| 40 | 5 | 15 | 77.8 | 70 |
| 41 | 4 | 15 | 90.49 | 51.84 |
| 42 | 6 | 14 | 88.31 | 84.7 |
| 43 | 4 | 19 | 97.94 | 90.09 |
| 44 | 5 | 19 | 74.4 | 92.18 |
| 45 | 4 | 15 | 79.53 | 64.15 |
| 46 | 4 | 19 | 74.74 | 74.01 |
| 47 | 5 | 15 | 79.72 | 75.64 |
| 48 | 4 | 15 | 76.76 | 68.44 |
| 49 | 4 | 15 | 92.97 | 95.55 |
| 50 | 4 | 15 | 70.2 | 63.48 |
| 51 | 5 | 15 | 86.31 | 68.65 |
| 52 | 5 | 15 | 86.69 | 85 |
| 53 | 5 | 15 | 76.27 | 66.54 |
| 54 | 3.5 | 13 | 74.98 | 85.88 |
| 55 | 6 | 18 | 93.92 | 71.99 |
| 56 | 5 | 15 | 75.84 | 88.26 |
| 57 | 5 | 15 | 82.75 | 81.63 |
