# Supplementary material for: Effects of the Vertebral Artery Ostium/Subclavian Artery Angle on In-Stent Restenosis after Vertebral Artery Ostium Stenting
Source: Biomed Res Int. 2021 Apr 27;2021:5527988. doi: 10.1155/2021/5527988 (PMC8101481; doi:10.1155/2021/5527988)
Supplement: Supplementary 4 — Clinical characteristics of 57 patients (3). [file 5527988.f4.docx]

Clinical Characteristics of 57 Patients (3)

| No | stenosis(%) | residual stenosis(%) | Symptoms  (follow up) | | restenosis |  |
| --- | --- | --- | --- | --- | --- | --- |
|  |  |  |  |  |  | Follow-up time(m) |
| 1 | 87.5 | 5.9 | | 1 | 0 | 12 |
| 2 | 78.8 | 4.9 | | 0 | 0 | 18 |
| 3 | 73 | 14.7 | | 0 | 0 | 12 |
| 4 | 71.7 | 24.6 | | 1 | 0 | 15.6 |
| 5 | 86.4 | 13.3 | | 0 | 0 | 18 |
| 6 | 83.1 | 19.1 | | 0 | 0 | 18 |
| 7 | 73.7 | 27.9 | | 0 | 0 | 6 |
| 8 | 70.6 | 34.5 | | 0 | 0 | 12 |
| 9 | 74.7 | 0 | | 0 | 0 | 12 |
| 10 | 82.5 | 33.3 | | 0 | 0 | 12 |
| 11 | 88.9 | 18.5 | | 1 | 0 | 24 |
| 12 | 74.6 | 17.7 | | 0 | 0 | 24 |
| 13 | 71.3 | 16.7 | | 0 | 0 | 18 |
| 14 | 70 | 7.5 | | 1 | 0 | 14.4 |
| 15 | 84.1 | 19.4 | | 1 | 0 | 16.8 |
| 16 | 77.2 | 14.5 | | 0 | 0 | 10.8 |
| 17 | 76.2 | 29.5 | | 0 | 0 | 15.6 |
| 18 | 73.7 | 6.7 | | 0 | 0 | 12 |
| 19 | 74.3 | 2.5 | | 0 | 0 | 12 |
| 20 | 85.3 | 2.4 | | 0 | 0 | 12 |
| 21 | 61.6 | 10.6 | | 0 | 0 | 12 |
| 22 | 87 | 26.5 | | 0 | 0 | 12 |
| 23 | 72.3 | 23.3 | | 0 | 0 | 12 |
| 24 | 70.2 | 1.5 | | 0 | 0 | 12 |
| 25 | 77 | 20.4 | | 1 | 0 | 18 |
| 26 | 79.9 | 10.7 | | 0 | 0 | 9.6 |
| 27 | 72 | 14 | | 0 | 0 | 6 |
| 28 | 77.7 | 19.1 | | 0 | 0 | 6 |
| 29 | 92.2 | 0 | | 1 | 0 | 9.6 |
| 30 | 70.9 | 14.4 | | 1 | 0 | 12 |
| 31 | 76.5 | 15.9 | | 0 | 0 | 12 |
| 32 | 70.4 | 9.3 | | 0 | 0 | 12 |
| 33 | 78.7 | 21 | | 1 | 0 | 12 |
| 34 | 85.2 | 9.8 | | 1 | 0 | 12 |
| 35 | 71.8 | 13.5 | | 0 | 0 | 12 |
| 36 | 81.8 | 0.9 | | 1 | 0 | 12 |
| 37 | 77 | 25.4 | | 1 | 0 | 12 |
| 38 | 73.3 | 2 | | 0 | 0 | 6 |
| 39 | 76.5 | 22.9 | | 0 | 0 | 12 |
| 40 | 75.7 | 20 | | 1 | 0 | 12 |
| 41 | 70.4 | 11.6 | | 1 | 0 | 12 |
| 42 | 81 | 2.9 | | 0 | 0 | 12 |
| 43 | 80.6 | 10.2 | | 1 | 0 | 12 |
| 44 | 80.2 | 6.6 | | 0 | 0 | 9.6 |
| 45 | 83.7 | 6.9 | | 1 | 0 | 12 |
| 46 | 83.7 | 7.9 | | 0 | 0 | 9.6 |
| 47 | 83.1 | 18.2 | | 1 | 0 | 12 |
| 48 | 78.7 | 1.9 | | 0 | 0 | 12 |
| 49 | 75.5 | 12.5 | | 0 | 0 | 12 |
| 50 | 78.9 | 18.5 | | 1 | 0 | 12 |
| 51 | 74.1 | 3.1 | | 0 | 0 | 24 |
| 52 | 73.6 | 17 | | 1 | 1 | 16.8 |
| 53 | 73.2 | 18.4 | | 0 | 1 | 21.6 |
| 54 | 71 | 13.7 | | 0 | 1 | 12 |
| 55 | 84.6 | 23.7 | | 1 | 1 | 12 |
| 56 | 70.6 | 9.3 | | 0 | 1 | 6 |
| 57 | 70.2 | 0 | | 0 | 1 | 15.6 |
